# Simulation-Based Peer Feedback Module for Pediatric Rapid Response Team Handoffs

**DOI:** 10.15766/mep_2374-8265.11544

**Published:** 2025-09-05

**Authors:** Rachael Herriman, Priti Jani, Nehal Patel, Brett Palama, Anu Elangovan

**Affiliations:** 1 Pediatric Emergency Medicine Fellow, Department of Pediatrics, University of California, San Francisco; 2 Associate Professor, Department of Critical Care Medicine, University of Chicago; 3 Associate Professor, Department of Pediatric Emergency Medicine, University of Chicago; 4 Associate Professor, Department of Pediatric Hospital Medicine, University of Chicago; 5 Neonatology Fellow, Department of Pediatrics, University of Chicago

**Keywords:** Simulation, Pediatrics, Rapid Response, Mentoring/Coaching, Handoffs, Competency-Based Medical Education (Competencies, Milestones, EPAs), Communication Skills

## Abstract

**Introduction:**

Deterioration of pediatric patients outside the PICU increases morbidity and mortality. Effective communication during rapid response team (RRT) events is essential. Although frameworks like SBAR (Situation, Background, Assessment, Recommendation) and ABC (Airway, Breathing, Circulation) exist, standardized RRT training for residents remains limited. This educational innovation integrates simulation, peer feedback, and deliberate practice to enhance resident communication, confidence, and handoff skills.

**Methods:**

Forty-four (52%) of 84 eligible pediatrics and internal medicine/pediatrics residents participated in a simulation-based module in 2022 prior to their pediatric inpatient rotation; 35 completed full training and surveys. The module included a baseline simulation, targeted instruction, practice scenarios with peer feedback, and a final simulation. Objectives focused on ABC-SBAR use, data synthesis, and increased confidence in leading RRTs. Pre- and postmodule surveys assessed knowledge and confidence. Faculty, who were masked to the participant's identity, measured ABC-SBAR handoff scores using audio recordings; peer evaluators also scored in-person simulations.

**Results:**

ABC-SBAR scores improved significantly (mean 5.1/12 to 9.3/12 pre- to postmodule, *p* < .001), with 32% of participants after training versus 5% before training reporting high confidence in leading a pediatric RRT (*p* < .001). Residents with no prior RRT experience showed the most significant gains. More than 50% of participants believed the training would improve real-world RRT performance.

**Discussion:**

This simulation-based module improved structured communication and leadership skills for pediatric RRT events. The approach is scalable and adaptable across training sites to enhance resident preparedness and patient safety.

## Educational Objectives

By the end of this activity, learners will be able to:

1. Demonstrate the use of the ABC-SBAR framework (Airway, Breathing, Circulation and Situation, Background, Assessment, Recommendation framework) to organize and communicate patient information during rapid response team (RRT) handoffs, achieving a postmodule score of at least 8/12 on the RRT assessment tool.
2. Apply peer feedback techniques to provide constructive critiques of a colleague's communication during RRT simulations, incorporating at least two areas of improvement in subsequent practice sessions.
3. Increase confidence in leading a pediatric RRT by self-reporting a minimum of 30% improvement in confidence levels on postmodule surveys.
4. Synthesize clinical observations, vital signs, and patient history to create a succinct, prioritized care plan during a simulated emergency handoff.

## Introduction

Clinical deterioration of pediatric patients outside the PICU is a critical issue associated with increased morbidity and mortality.^1^ Effective communication and teamwork during rapid response team (RRT) events are essential to mitigating such deterioration, yet these skills are often inconsistent among medical trainees.^2^ At our tertiary care children's hospital, RRTs can be requested by any staff member concerned about a patient's condition, providing an opportunity for early intervention. During these RRT events, pediatric residents are tasked with communicating with intensive care fellows to assess the patient's clinical status and determine interventions to prevent further decline. However, the processes and coordination of medical handoffs vary significantly among trainees, which can compromise the quality of care provided during these critical moments.^3,4^

Miscommunication is a leading cause of adverse events in health care. Up to 80% of serious medical errors are attributed to failures in communication during patient handoffs.^5^ The WHO Patient Safety Council emphasizes that improving communication among health care providers is essential to reducing millions of preventable adverse events globally.^6^ Furthermore, the ACGME mandates that residency programs teach and evaluate handoff quality, underscoring the importance of structured communication training.^7^ Frameworks such as SBAR (Situation, Background, Assessment, Recommendation) and ABC (Airway, Breathing, Circulation) in conjunction with SBAR (ABC-SBAR) provide systematic approaches for handoff communication.^2,8–10^ Despite these frameworks, curricula often lack opportunities for repeated, structured practice and feedback, highlighting the need for simulation-based approaches grounded in deliberate practice, a structured and purposeful form of practice aimed at improving specific aspects of performance through focused effort, feedback, and repetition.

The target audience for this educational innovation includes pediatrics residents and internal medicine/pediatrics residents at various training levels (PGY 1–4). Despite prior exposure to RRTs, many residents lack the confidence, communication skills, and structured approach required to lead these high-stakes scenarios. Research, such as the findings reported by McCrory and colleagues, reveals that while handoff training can be effective, opportunities for reinforcement are limited—underscoring the importance of repeated practice and peer feedback in skill development.^11^ For instance, only 29% of residents surveyed in that study recalled the ABC-SBAR framework 2.5 years after training. These findings highlight a significant gap in the literature and the need for innovative approaches to improve retention and applicability of these critical skills.

Simulation has proven to be an effective instructional method for teaching high-risk, low-frequency clinical skills.^12–14^ It allows learners to engage in realistic, high-pressure scenarios in a controlled and safe environment. By integrating peer-to-peer feedback and repetitive practice into simulation-based training, our module builds on prior work and aligns with recommendations for experiential learning and feedback-driven improvement.^15^

This module represents a novel contribution by combining simulation-based learning, peer feedback, and utilization of a structured-repetition ABC-SBAR framework as a structured communication tool, further ensuring clarity and prioritization during RRT patient handoffs. These features address previously identified limitations in similar curricula and provide a sustainable, reproducible model for enhancing pediatric RRT training.

## Methods

This educational activity was developed collaboratively by our pediatric emergency medicine faculty and medical education specialists using principles of deliberate practice and experiential learning, and we situated it within the residency simulation curriculum to enhance communication during rapid response scenarios.

### Training and Implementation

Before we implemented this simulation-based RRT peer feedback training module, we conducted preparatory training for faculty facilitators and senior residents via a conversational Zoom meeting and an in-person module walkthrough. This module covered delivery logistics, simulation tools, and use of the RRT scoring tool. While we did not conduct formal large-group training, our faculty had prior experience with the material and simulation-based instruction, supporting consistent and informed facilitation. We engaged trained senior residents to serve as peer evaluators during the simulation, but they did not participate as learners. We did not require additional specialized training for participating residents. We used an instructor guide (Appendix A) to support facilitators in guiding the overall module.

We conducted the simulation module before the pediatrics residents and dual internal medicine/pediatrics residents started their general inpatient rotations at the University of Chicago Comer Children's Hospital, thereby ensuring that the residents had foundational communication and leadership skills before encountering real RRT events. Participants self-selected during their inpatient rotation assignments. Before participating in the simulation, residents completed the premodule survey sent via email (Appendix B). To minimize potential bias, participants completed this survey before receiving instructional materials.

We divided residents into groups of 2–6 learners, with a preceptor-to-learner ratio of 1:2, allowing for individualized feedback and close observation throughout the module. Facilitators consisted of trained pediatric faculty physicians and simulation center staff, who guided residents through each stage of the training.

### Module Structure

We began the 90-minute module with a 5- to 10-minute prebrief and consent process, during which we introduced participants to the module objectives, structure, and expectations. The baseline simulation lasted 20 minutes, where participants were given 2 minutes to review the scenario prompt, followed by 5 minutes to communicate with a simulated bedside nurse and assess the patient before the arrival of the simulated PICU fellow (a facilitator). Once ready, they notified the facilitator to begin an audio-recorded handoff presentation, which lasted approximately 2 minutes before transitioning to the next participant. We utilized two simulation rooms for handling of the first simulated case (Case 1, Appendix C), with one facilitator each, to facilitate efficiency.

Following the baseline simulation, we led a 10-minute debrief and didactic session and provided participants with immediate feedback on their performance. Facilitators guided discussions using the ABC-SBAR framework and handout, reinforcing effective communication strategies and structured handoff techniques. We distributed printed handouts (Appendix D) to supplement their learning. Next, residents engaged in 20 minutes of practice scenarios, applying the framework in real-time during two simulated cases (Cases 2 and 3, Appendices E and F). These cases allowed for peer and facilitator feedback, helping residents refine their communication skills in a lower-stakes setting without requiring a full simulation setup.

The module then progressed to a final simulation block lasting 20 minutes, where participants demonstrated their communication skills in a structured environment utilizing the RRT scoring tool (Appendix G). In the final block, two simulated cases (Case 4 and Case 5, Appendices H and I) were used, where each participant had 2 minutes to review the scenario prompt, followed by 5 minutes to communicate with a simulated bedside nurse and assess the patient before the arrival of the PICU fellow. The module culminated with a 2-minute recorded presentation of the ABC-SBAR framework, allowing direct comparison with baseline performance. For the final scenario, participants were scored by peer evaluators via a real-time phone survey and audio recording, with the results later analyzed.

To conclude the module, we asked participants to spend the final 10 minutes completing surveys that assessed their confidence levels, perceived skill improvement, and the usability of the RRT tool (Appendix J). We used any remaining time for a short debriefing, allowing residents to reflect on their experiences by identifying strengths and areas for improvement. This reflection period also allowed participants to offer constructive feedback to refine and enhance future iterations of the module.

### Assessment and Evaluation

We used a multimodal evaluation strategy to assess the module's effectiveness. Pre- and postmodule surveys measured changes in residents’ confidence levels, familiarity with the ABC-SBAR framework, and preparedness for leading pediatric RRTs (Appendices B and J). Additional questions evaluated the clarity and usability of the RRT tool. The pre- and postmodule surveys were developed by pediatric emergency medicine faculty and medical education specialists to align with the module's learning objectives. Items were informed by key domains from prior literature on handoff communication and simulation training, including constructs from previously published studies, such as that used in the study by McCrory and colleagues.^10^ However, the survey itself was not adapted from a validated instrument.

We assessed structured communication performance using the RRT Pediatric Scoring Tool (Appendix G), adapted from McCrory and colleagues.^10^ The scoring tool included 16 items, each worth 1 point. Items 1a-1d included evaluation of vital signs, laboratory results, intake, output, and eCART. Patient information was obtained by a mock electronic medical record (EMR) patient interface. We adapted the tool from McCrory et al. by adding eCART evaluation. eCART is a numeric designation unique to our institution—an algorithm that uses vital signs, laboratory results, and demographic data to calculate a real-time risk score of clinical decompensation.

In comparisons between direct audio recording and in-person ABC-SBAR performance scores, we excluded visual observation items 1a-1d, resulting in a maximum score of 12 points. Higher scores indicated greater proficiency. Performance metrics included changes in ABC-SBAR scores, self-reported confidence, and participant feedback. Peer evaluators provided additional scoring and constructive feedback on communication and leadership. We did not conduct in-person peer scoring during the baseline or practice scenarios; peer evaluations were limited to the final simulation case to reduce bias and preserve the formative learning environment.

We assessed hand-off content on patient vital signs, airway, breathing, circulation, and organization. We encouraged residents to prioritize critical information within 10 seconds and complete handoffs within 30 seconds.

### Audio Recordings

Baseline and final simulation handoffs were independently scored by two trained simulation instructors, who were masked to the participant's identity and timing (pre/postmodule). We assessed inter-rater reliability using intraclass correlation coefficients (ICCs) based on a two-way random-effects model. We conducted additional statistical analyses, including Kruskal-Wallis tests, to compare performance metrics across different time points. These assessments provided a comprehensive evaluation of knowledge acquisition, skill development, and confidence improvements resulting from the training.

### Logistics and Environment

We conducted the training module in the University of Chicago Medicine simulation center, arranged to facilitate didactic instruction and simulation-based practice. We used a small conference room setup with a basic round-table seating arrangement for the initial didactic session, practice scenarios, and final reflections. We placed three laptops within the conference room with preloaded postmodule surveys to encourage participant completion and flow efficiency.

To simulate a realistic clinical environment, we utilized two additional rooms equipped with high-fidelity mannequins and basic monitoring tools, including EKG leads, a blood pressure cuff, a pulse oximeter, and a vital sign monitor. We changed room setup following the Case 1 simulation to facilitate the final case scenarios.

Participants had access to a nasal cannula, IV fluids, procedural equipment, stethoscopes, and medication vials to enhance realism in patient management exercises. Additionally, we provided a computer with EMR access and a printed eCART sheet nearby to simulate real-time clinical decision-making. We used audio recording equipment to capture resident performance for evaluation purposes.

Facilitators and simulation center staff ensured that all required materials, including printed scoring sheets (Appendix G) and instructional guides, were prepared 2 weeks in advance to facilitate seamless implementation of the module.

This innovation was deemed exempt from institutional review board review (IRB21-1571) by the University of Chicago Institutional Review Board, as it was classified as an educational initiative involving minimal risk to participants.

## Results

Forty-four of 84 eligible pediatrics and internal medicine/pediatrics residents participated in the module, with 35 completing all required components, including both the pre- and postmodule surveys and the full module. Nine residents were unable to complete the full training due to clinical duties. Simulation cases were implemented for five individual training module iterations with residents, dependent on resident clinical schedules and availability. Materials were utilized by three different faculty members with expertise in pediatric critical care, emergency medicine, hospital medicine, and simulation-based education. Pilot testing revealed challenges in balancing simulation timing with in-depth debriefing. No significant technical or content-related barriers were encountered during implementation.

Forty-one percent of the 44 participating residents were in their first year of training (Table). More than 50% of the participants had previously led an RRT, 95% reported previous RTT exposure, and 12% had health care experience before residency.

**Table.**
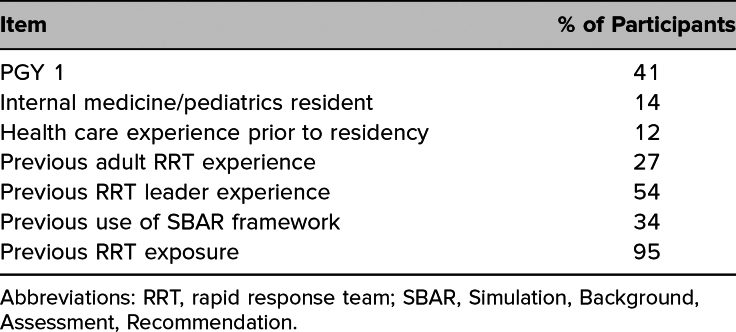
Characteristics of Participants in the Pediatric RRT Simulation Module (*N* = 44)

**Figure 1.**
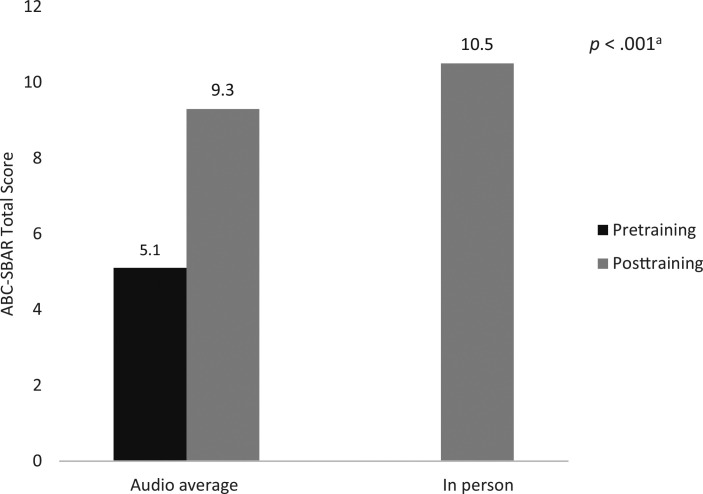
Total scores, pre- and posttraining, rating resident use of the ABC and SBAR framework in pediatric rapid response team simulations, including comparison of mean evaluator scores of audio recordings (two raters) and resident in-person performance. Abbreviations: ABC, Airway, Breathing, Circulation; SBAR, Situation, Background, Assessment, Recommendation. ^a^Comparison between pre- and posttraining by paired *t* test.

Audio recording total scores for participant performance of the ABC-SBAR framework improved significantly from pre- to postmodule (mean score, 5.1/12 premodule vs. mean 9.3/12 postmodule, *p* < .001; Figure 1). In comparison, in-person total ABC-SBAR peer scores averaged 10.5/12 after training. Inter-rater reliability of scores between modalities was low (ICC = 0.27).

The total time of presentations significantly increased compared with those before training, and the average times had broad standard deviations (audio recordings, mean 64.5 seconds [SD = 23.5] pretraining vs. 93.5 seconds [SD = 32] posttraining, *p* < .001; in-person, mean 99.0 seconds [SD = 35.6] posttraining). Thus, the average total reported time was 6 seconds longer when peer evaluators rated the residents in person.

**Figure 2.**
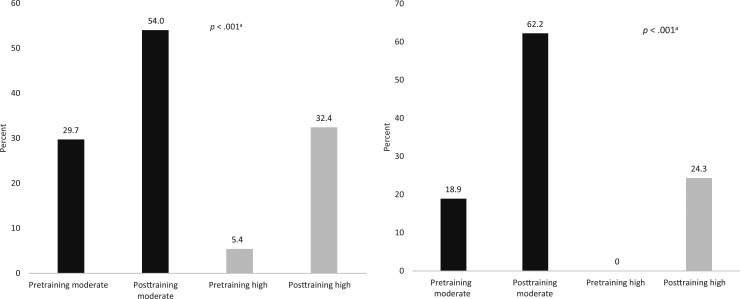
Resident self-reported confidence levels, pre- and posttraining, in leading a pediatric rapid response team (left panel) and utilizing the ABC and SBAR frameworks (right panel) pre- and post-RRT training, stratified by moderate and high confidence levels. Abbreviations: ABC, Airway, Breathing, Circulation; SBAR, Situation, Background, Assessment, Recommendation. ^a^Comparison between pre- and posttraining by paired *t* test and Wilcoxon signed-rank test.

In addition to objective scoring, self-reported confidence levels (rated on a scale of 0–5; 1 = *strongly disagree*, 5 = *strongly agree*) were assessed by participants before and after training. Resident confidence in both leading an RRT and utilizing the ABC-SBAR tool improved significantly following the training (Figure 2). Prior to the module, only 5% of participants reported high confidence in leading an RRT, whereas 32% of participants reported high confidence posttraining (*p* < .001). No participants reported no or low confidence in leading an RRT.

**Figure 3.**
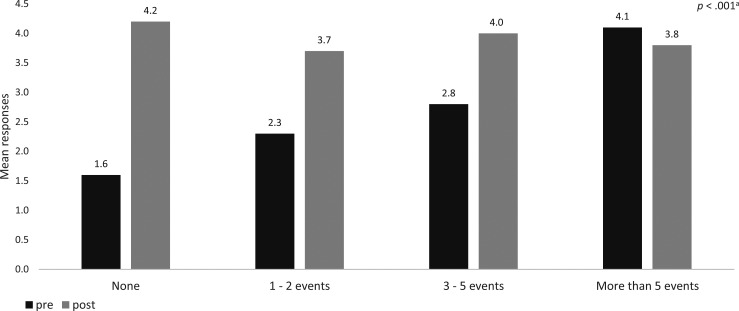
Resident self-reported preparedness, pre- and posttraining, to lead an RRT, stratified by previous RRT experience (number of events with prior participation). Abbreviation: RRT, rapid response team. ^a^Comparison between pre- and posttraining by paired *t* test and Wilcoxon signed-rank test.

Resident preparedness to lead an RRT (rated on a scale of 0–5; 1 = *strongly disagree*, 5 = *strongly agree*) significantly improved following the module (*p* < .001), with the most significant gains observed among those with no prior RRT experience (mean score 1.6 pretraining vs. 4.2 posttraining) (Figure 3). Residents with limited experience (1–2 prior RRT events) also demonstrated substantial improvement in preparedness (mean score 2.3 pretraining vs. 3.7 posttraining), while those with greater exposure (3–5 or >5 prior RRT events) showed smaller but still positive shifts in self-reported preparedness after the training. The most significant improvement in preparedness occurred among those with the least prior RRT experience. Most residents also strongly agreed that they had received adequate training to lead an RRT following participation (*p* < .001).

PGY level did not significantly impact improvements in confidence or preparedness to lead an RRT following training (all *p* > .40). On the surveys, 57% of residents agreed and 22% strongly agreed that training would improve patient quality of care during a pediatric RRT event.

## Discussion

This simulation-based module improved communication skills and confidence among pediatrics residents and internal medicine/pediatrics residents during simulated RRT events. Structured simulation training, peer feedback, and deliberate practice proved to be effective educational strategies for addressing gaps in RRT training. Significant gains in ABC-SBAR scores and participant confidence reflect the module's alignment with Kirkpatrick level 1 (reaction) and level 2 (learning).^16^ Participants reflected positively on the module's value, though satisfaction was not formally measured.

Several lessons emerged during the development and implementation of this module. Refinements to the scoring guidelines improved participants’ understanding of the ABC-SBAR framework, facilitating more objective evaluations. Peer-to-peer feedback was particularly impactful, fostering bidirectional learning in a safe and collaborative environment. Structured debriefing sessions further enhanced skill development by encouraging reflection and identification of areas for growth. These strategies helped residents develop the confidence to lead RRTs effectively, particularly those with minimal prior experience.

While this module demonstrated improvements in resident confidence, structured communication, and preparedness for RRT leadership, several limitations should be considered. First, this innovation was conducted at a single institution, so its findings may not be fully generalizable to other settings. Second, participants’ varying levels of prior RRT experience necessitated content adjustments to ensure inclusivity, potentially limiting applicability to more homogeneous learner groups. Third, while the structured simulation cases effectively enhanced skills, they did not fully replicate the complexity and cognitive demands of real-life RRT scenarios. Incorporating higher-intensity cases with greater cognitive load could better simulate the pressures of actual emergencies. Fourth, the absence of direct patient outcome data prevents assessment of the broader clinical impact of the training. While participants perceived the training as beneficial, objective evidence of improved patient care (Kirkpatrick level 4) during actual RRT events was not collected. Fifth, time constraints limited the depth of debriefing sessions, which may have reduced opportunities for reflective learning and discussion. Sixth, variability in peer scoring reliability presented another potential limitation. Although peer evaluators used a standardized rubric, they were not masked to participant identity, and we did not formally assess for interpersonal bias. The low inter-rater reliability (ICC = 0.27) suggests inconsistent scoring between peers and faculty raters, which may reflect differences in perspective or comfort with evaluation. While facilitators provided briefing on scoring guidelines, additional peer evaluator training or score calibration may be needed to enhance consistency in future implementations. While peer feedback was embedded as a core component of the module, we did not include a formal evaluation of its quality. The usefulness, accuracy, and impact of peer-delivered feedback remain areas for further study. Finally, formal pilot testing prior to survey use was not completed. This represents a limitation of the current evaluation. Future iterations of the module may incorporate structured survey validation, including cognitive interviewing or expert review, to improve instrument reliability and interpretability. These limitations highlight areas for future investigation, including improving rater consistency, evaluating peer feedback quality, integrating longitudinal assessments, and exploring real-time clinical impact.

For this educational innovation, we utilized our medical institution's EMR system to enter mock patient cases and assessment items 1a-1d of the scoring tool. We acknowledge that the use of eCART is specific to our institution, which may limit the generalizability of our findings. We excluded items 1a-1d from our data analysis to ensure direct comparability of in-person and audio recording ratings of participant performance. Individual institutions can tailor these items as needed to align with their specific systems and practices.

Future iterations of this module should focus on several key enhancements. Long-term follow-up studies are needed to assess knowledge retention and evaluate the module's impact on real-world patient outcomes during RRT events. Incorporating more complex scenarios and interprofessional team participation would improve realism and applicability. Expanding the program to additional residency programs and institutions could enhance its generalizability, and integrating faculty development resources would ensure consistent implementation and evaluation. Providing additional time for reflection and debriefing during the training would further enrich the learning experience.

By addressing these areas, this module has the potential to become a scalable and sustainable model for improving RRT training. Future research should explore its impact on patient safety and outcomes, offering valuable insights for broader educational policy and curriculum development.

This module addresses a critical gap in pediatric RRT training by equipping residents with the tools and confidence to lead effectively in high-pressure situations. The combination of structured simulation training, peer-to-peer feedback, and deliberate practice has demonstrated significant improvements in communication, confidence, and leadership skills. These findings provide a strong foundation for broader implementation and highlight the potential of simulation-based training to enhance patient safety and outcomes.

## Appendices


RRT Facilitator Guide.docxRRT Premodule Questions.docxCase 1.docxRRT Handout.docxCase 2.docxCase 3.docxRRT Scoring Tool.docxCase 4.docxCase 5.docxRTT Postmodule Questions.docx
*All appendices are peer reviewed as integral parts of the Original Publication.*
