# Supplementary material for: Simulation-Based Peer Feedback Module for Pediatric Rapid Response Team Handoffs
Source: MedEdPORTAL. 2025 Sep 5;21:11544. doi: 10.15766/mep_2374-8265.11544 (PMC12411645; doi:10.15766/mep_2374-8265.11544)
Supplement: Supplementary file 1 — RRT Facilitator Guide.docxRRT Premodule Questions.docxCase 1.docxRRT Handout.docxCase 2.docxCase 3.docxRRT Scoring Tool.docxCase 4.docxCase 5.docxRTT Postmodule Questions.docx [file mep_2374-8265.11544-s001.zip › A. RRT Facilitator Guide.docx]

**Appendix A: Facilitator Guide for Simulation-Based Peer-to-Peer Coaching Curriculum**

**Facilitator Use Instructions:**

This guide is intended for faculty facilitators implementing the RRT simulation-based training module. It should be used during the live instructional session to guide facilitators through each stage of the 90-minute curriculum, including the pre-brief, baseline simulation, didactic overview, practice cases with peer feedback, final simulation, and post-session surveys. Facilitators should review this document prior to the session and have it available throughout the simulation for reference.

**Introduction**

Welcome to the Simulation-Based Peer-to-Peer Coaching Curriculum for Pediatric Rapid Response Team (RRT) Handoffs. This guide provides facilitators with detailed instructions to deliver the training effectively, ensuring participants achieve the outlined educational objectives.

**Purpose**

The purpose of this curriculum is to focus on the efficiency, confidence, and communication of residents in RRT scenarios. While clinical knowledge and synthesis of the primary concern based on clinical evaluation are important, this training emphasizes communication skills during high-pressure situations. Prior to simulation, residents will complete a pre-simulation assessment (Appendix I).

**Educational Objectives**

By the end of this activity, learners will:

1. Demonstrate the use of the ABC-SBAR framework to organize and communicate patient information during RRT handoffs, achieving a post-simulation score of at least 8/12 on the ABC-SBAR assessment tool.
2. Apply peer feedback techniques to provide constructive critiques of a colleague’s communication during RRT simulations, incorporating at least two areas of improvement in subsequent practice sessions.
3. Increase confidence in leading a pediatric RRT by self-reporting a minimum of a 30% improvement in confidence levels on post-intervention surveys.
4. Synthesize clinical observations, vital signs, and patient history to create a succinct, prioritized care plan during a simulated emergency handoff within a two-minute timeframe.

**Session Agenda and Facilitator Instructions**

**1. Pre-Brief and Consent (5–10 minutes)**

- **Objective**: Introduce the session objectives, structure, and expectations while obtaining participant consent.
- **Facilitator Actions**:
  - Welcome participants and introduce facilitators.
  - “During rapid response team (RRT) events, clear and effective communication is crucial for ensuring patient safety and preventing further deterioration. These scenarios often involve high-pressure decision-making and require a structured approach to ensure that critical information is accurately conveyed. Miscommunication during these moments can lead to adverse outcomes, which is why this training focuses on equipping you with tools like ABC-SBAR. With practice, you’ll build confidence and efficiency in delivering concise, organized handoffs that improve team coordination and patient care.”
  - A brief discussion of resident prior handoff experience, challenges, if any.
  - Provide a brief overview of the session agenda and educational objectives.
  - Distribute Appendix B materials for participant reference.
  - Ensure consent forms are completed.
  - Introduce the setup of the patient room, including the eCART sheet, EPIC playground, and patient vitals posted on the wall. Review available monitoring tools (e.g. blood pressure cuff, stethoscope, nasal cannula)

**2. Baseline Simulation (20 minutes)**

- **Objective**: Assess participants' baseline RRT handoff performance.
- **Facilitator Actions**:
  - With the second facilitator, determine room assignments for Case 1.
  - Provide each participant with a scenario prompt (Appendix C).
  - Allow participants 5 minutes to review the scenario, assess the patient, and prepare their presentation.
  - Facilitate a simulated handoff with each participant presenting for 2 minutes. Ensure audio recording for later review.
  - Allocate ~2 minutes to transition between participants. Each facilitator will oversee 1-3 participants, depending on group size.

**3. Initial Debrief and Didactics (10 minutes)**

- **Objective**: Provide feedback on baseline simulation performance and introduce the ABC-SBAR framework (Appendix B).
- **Facilitator Actions**:
- Initiate a structured reflection by asking participants to identify one strength and one area for improvement from their baseline simulation.
- Provide targeted feedback on communication clarity, organization, and critical thinking using the ABC-SBAR framework.
- Distribute the ABC-SBAR information sheet and facilitate a brief discussion on how structured communication improves patient safety and team coordination.

**4. Resident Pair Simulations (20 minutes)**

- **Objective**: Allow participants to apply the ABC-SBAR framework in practice scenarios.
- **Facilitator Actions**:
  - Provide participants with Cases 2 and 3 (Appendices D and E) Simulation rooms/mannequins are not required for this portion.
  - Assign residents into pairs and provide the RRT communication scoring tool (Appendix F) for use during evaluations.
  - Facilitate two practice rounds for each case, with residents alternating between presenting and observing roles.
  - Encourage observers to use the scoring sheet to provide constructive feedback. If an odd number of participants, assign the extra resident to practice with the facilitator or a pair.

**5. Final Simulation (20 minutes)**

- **Objective**: Evaluate participants' performance after training.
- **Facilitator Actions**:
  - Provide participants with the final scenarios (Appendices G Case 4 and H Case 5). Assign each participant one of the final scenarios (Appendices G or H) for evaluation.
  - Allow 2 minutes for participants to review the prompt and 5 minutes to assess the patient and communicate with the bedside nurse.
  - Facilitate a simulated handoff, ensuring audio recording of each participant’s 2-minute presentation.
  - Ensure observing residents use the scoring sheet to evaluate their peers, including timing-specific components (e.g., parts 12 and 13 of the scoring tool).
  - Assign different cases to each pair for the final simulation.

**6. Final Debrief and Surveys (10 minutes)**

- **Objective**: Collect participant feedback and provide closure to the session.
- **Facilitator Actions**:
  - Lead a group discussion, asking residents to share one thing they learned and one area they wish to continue improving.
  - Address any remaining questions or concerns.
  - Provide participants with a QR code or web link to complete the post-session survey (Appendix J).
  - Conclude the session by thanking participants and offering additional resources for continued learning.

**Logistics and Materials**

- **Room Setup**: Arrange a small conference room for the didactic, case practice, and debriefing components, and two simulation rooms equipped with high-fidelity mannequins for baseline and final scenarios.
- **Equipment**:
  - High-fidelity mannequins with basic monitoring tools (EKG leads, blood pressure cuffs, pulse oximeters, and vital sign monitors).
  - Procedural tools, including nasal cannulas, IV fluids, stethoscopes, and medication vials.
  - Audio recording equipment for handoff assessments.
  - Computers with electronic medical record (EMR) access and eCART sheets.
- **Handouts**:
  - Appendix B: ABC-SBAR framework
  - Appendix F: Scoring sheet for peer evaluations

Thank you for facilitating this curriculum. Your role is essential in helping participants develop the confidence and skills needed to lead pediatric RRTs effectively.
