# Supplementary material for: Simulation-Based Peer Feedback Module for Pediatric Rapid Response Team Handoffs
Source: MedEdPORTAL. 2025 Sep 5;21:11544. doi: 10.15766/mep_2374-8265.11544 (PMC12411645; doi:10.15766/mep_2374-8265.11544)
Supplement: Supplementary file 1 — RRT Facilitator Guide.docxRRT Premodule Questions.docxCase 1.docxRRT Handout.docxCase 2.docxCase 3.docxRRT Scoring Tool.docxCase 4.docxCase 5.docxRTT Postmodule Questions.docx [file mep_2374-8265.11544-s001.zip › B. RRT Premodule Questions.docx]

**Rapid Response Team Pre-Assessment**

Identifier (Please use the first letter of your first name, the day of birth [e.g., Feb. 7 would be 07], and the first letter of your hometown):

1. What level PGY are you?
2. PGY1
3. PGY2
4. PGY3
5. PGY4
6. PGY5
7. Please indicate your residency program.
   1. Pediatrics
   2. Internal Medicine/ Pediatrics
8. Prior to becoming a pediatric resident, did you work as another type of healthcare provider? Yes/No
   1. If yes, Please, select any or all of the following specialties in which you have worked as a healthcare provider? Physician, Nurse Assistant, Nurse, EMT/Paramedic, Firefighter, RT
9. How many RRT events have you participated in previously: **0, 1, 2, 3, 4, 5, >5?**
10. How many Adult RRT events have you participated in previously: **0, 1, 2, 3, 4, 5 >5?**
11. How many times have you been the leader of a RRT event? **0, 1, 2, 3, 4, 5 >5**
12. How many times have you used SBAR communication during a handoff? **0, 1, 2, 3, 4, 5 >5**
13. Which of the following below appropriately describes SBAR?
14. Situation, Baseline, Assessment, Referral
15. An acronym to provide structure in explaining a patient’s current status and background as well as a provider’s assessment and recommendation for what they would like to see happen for the patient
16. Sample, Background, Action, Repeat
17. An outline for a plan to transfer a patient in distress to an intensive care unit
18. Where is the appropriate location to gather (PICU and Floor team) during a RRT?
19. In the hallway outside the patient’s room
20. At a patient’s bedside
21. At the nursing station
22. In the inpatient floor team room
23. What members of the care team are supposed to be present at a RRT? Please select all that apply.
24. Any resident on the assigned inpatient team
25. Hospitalist attending
26. Nurse
27. PICU fellow
28. Respiratory Therapist
29. PICU Resident
30. General floor charge nurse
31. Senior resident
32. Who leads the RRT? Please choose one.
33. Hospitalist attending
34. Any resident on the assigned inpatient team
35. The nurse, if they call the RRT
36. PICU fellow
37. Senior resident
38. I have led one or more RRT(s) in the past.
39. Yes
40. No
41. I have witnessed my senior or co-resident leading a RRT.
42. Yes
43. No
44. I have used the SBAR format when leading a RRT.
45. Yes
46. No
47. N/A
48. Please rate the following on a scale from 1-5, 1= strongly disagree 2=disagree 3= unsure 4 = agree 5 = strongly agree or if not applicable, please select N/A.
49. I feel confident to lead a RRT.
50. I feel well prepared to lead a RRT.
51. A successful RRT improves patient care.
52. I have received adequate training to lead a RRT.
