# Supplementary material for: Simulation-Based Peer Feedback Module for Pediatric Rapid Response Team Handoffs
Source: MedEdPORTAL. 2025 Sep 5;21:11544. doi: 10.15766/mep_2374-8265.11544 (PMC12411645; doi:10.15766/mep_2374-8265.11544)
Supplement: Supplementary file 1 — RRT Facilitator Guide.docxRRT Premodule Questions.docxCase 1.docxRRT Handout.docxCase 2.docxCase 3.docxRRT Scoring Tool.docxCase 4.docxCase 5.docxRTT Postmodule Questions.docx [file mep_2374-8265.11544-s001.zip › C. Case 1.docx]

**Instructions for Use**

This simulation case is designed to be used during a 90-minute rapid response training module for pediatric and medicine/pediatric residents (PGY 1–4). Facilitators should familiarize themselves with the case details in advance and use this guide to simulate the patient scenario, provide cues at designated time points, and prompt learners as needed. This case is intended to be used in combination with the ABC-SBAR communication handout (Appendix B) and the RRT scoring tool (Appendix F). Facilitators should guide learners through assessment, intervention, and structured handoff communication using the ABC-SBAR format, followed by a debrief session.

**CASE 1:**

| SIMULATION CASE TITLE | Rapid Response for HLH with Respiratory Distress |
| --- | --- |
| AUTHORS | Rachael Herriman, MD, Priti Jani, MD, MPH |
| LEARNER AUDIENCE | Pediatric and medicine/pediatric residents (PGY 1–4) involved in inpatient rotation |
| PATIENT NAME | Georgia |
| PATIENT AGE | 18 months |
| CHIEF COMPLAINT | Fever and tachycardia with respiratory distress |
| PHYSICAL SETTING | Pediatric inpatient unit transitioning to PICU |
| Brief Narrative Description of Case | Georgia is an 18-month-old female admitted for hemophagocytic lymphohistiocytosis (HLH) secondary to Epstein-Barr virus (EBV). She presents with persistent fever, tachycardia, and increased work of breathing. Learners must recognize clinical deterioration, communicate using the ABC-SBAR framework, and recommend PICU transfer with advanced respiratory support. |
| Primary Learning Objectives | 1. Recognize signs of clinical deterioration, including respiratory distress and tachycardia.  2. Synthesize patient history, physical exam findings, and vital signs to formulate a clinical assessment.  3. Demonstrate effective handoff communication using the ABC-SBAR framework.  4. Collaborate with the team to recommend PICU transfer and initiate advanced respiratory support. |
| Critical Actions | 1. Identify tachycardia, fever, and increased work of breathing.  2. Perform stabilization measures as needed (e.g., ensure oxygen delivery).  3. Deliver a structured patient handoff using ABC-SBAR.  4. Recommend PICU transfer and initiate advanced interventions. |
| Learner Preparation or Prework | 1. Review the ABC-SBAR communication framework.  2. Study the clinical presentation and management of HLH.  3. Familiarize with indications for ventilators |

| **Section** | **Details** |
| --- | --- |
| Initial Vital Signs | HR: 182, RR: 42, O2 saturation: 97% on 3L NC, BP: 83/46, Temp: 38.2 |
| Overall Setting and Appearance | Patient is lying in bed, appearing uncomfortable. Increased work of breathing noted with subcostal retractions. Central line and low flow nasal cannula in place. |
| Standardized Participants | Facilitator acting as nurse, stating: 'Hi, I’m covering for the nurse taking care of Georgia and am worried about her vital signs, she has a fever and is tachycardic.' |
| HPI | 18-month-old female with history of multiple wheezing-associated respiratory infection episodes, now believed to have HLH secondary to an EBV lymphoproliferative disorder. Persistent fevers and intermittent increased work of breathing. |
| Past Medical/Surgical History | Multiple wheezing episodes, suspected HLH secondary to EBV. |
| Medications | Decadron and Etoposide (chemotherapy regimens). |
| Allergies | Not specified. |
| Family History | Not specified. |
| Physical Exam - General | Lying in bed, uncomfortable appearing. |
| Physical Exam - Lungs | Increased work of breathing with subcostal retractions. |
| Physical Exam - Cardiovascular | RRR, tachycardia. |

Instructor Notes

| **Intervention / Time Point** | **Change in Case** | **Additional Information** |
| --- | --- | --- |
| 2 minutes into the case | BP begins decreasing if no intervention is made (e.g., fluids, oxygen). | Nurse alerts: 'Doctor, the blood pressure is now 90/45.' |
| Nursing expresses concern about vital signs | Tachycardia or desaturation may worsen. | RN or facilitator states: 'I’m worried because the heart rate is still increasing.' |
| Participant decides to administer medication or intervention | Patient’s vital signs stabilize or worsen depending on intervention accuracy and timing. | Improvement or deterioration provides real-time feedback for decisions. |
| Participant evaluates physical exam findings | Findings such as wheezing, retractions are clarified or worsen. | Patient may state: 'It’s harder to breathe' or 'That hurts!' depending on the context. |

Ideal Scenario Flow

The learners enter the room to find a toddler in respiratory distress with signs of hypoxia and tachycardia. They immediately place the patient on bedside monitors and administer supplemental oxygen. The vital signs are carefully monitored, and an IV fluid bolus is ordered due to concerns for hypotension. Despite initial interventions, the patient’s respiratory distress worsens. Learners recognize the need for escalation and perform a detailed physical examination, noting increased work of breathing and subcostal retractions. Learners consult the PICU team presenting a successful ABC-SBAR sign out.

Anticipated Management Mistakes

1. **Delay in initiating oxygen therapy:** Learners may not promptly recognize the need for supplemental oxygen despite hypoxia.
2. **Failure to escalate care in a timely manner:** Some learners might hesitate to initiate a PICU consult, delaying critical interventions.
3. **Incomplete physical examination:** Learners may miss signs of increased work of breathing and subcostal retractions, leading to an incomplete assessment.
