# Supplementary material for: Simulation-Based Peer Feedback Module for Pediatric Rapid Response Team Handoffs
Source: MedEdPORTAL. 2025 Sep 5;21:11544. doi: 10.15766/mep_2374-8265.11544 (PMC12411645; doi:10.15766/mep_2374-8265.11544)
Supplement: Supplementary file 1 — RRT Facilitator Guide.docxRRT Premodule Questions.docxCase 1.docxRRT Handout.docxCase 2.docxCase 3.docxRRT Scoring Tool.docxCase 4.docxCase 5.docxRTT Postmodule Questions.docx [file mep_2374-8265.11544-s001.zip › D. RRT Handout.docx]

**ABC SBAR Pediatric Emergency Response (RRT)**

**Learner Use Instructions:**

This handout should be provided to residents during the didactic portion of the RRT simulation training. It serves as a reference for how to structure an ABC-SBAR handoff and should be used during the practice scenarios and final simulation. Learners may also use this as a clinical reference during inpatient rotations.

This simulation activity is meant to better prepare you to assess an evolving inpatient pediatric situation. When we are able to communicate in a confident and effective way, we have the opportunity to provide better care for patients. We will be evaluating both the content and timing of the presentation during today’s simulation.

The purpose of ABC SBAR is to offer a standardized, simple outline to communicate the appropriate information during an RRT. Variations of this structure have been used in previous studies evaluating similar handoffs.

**A** Airway

**B** Breathing

**C** Circulation

**S** Situation

**B** Background

**A** Assessment

**R** Recommendation

Immediate assessment of these, same as if in a trauma.

What is the emergency?

What is the clinical background? What has been tried so far?

What do you think is causing this emergency?

What needs to be done next?

**What to do:**

Decide to call a RRT (ideally, you have discussed this

with nursing).

Await the arrival of the full RRT team (PICU fellow, PICU

charge nurse, respiratory therapist, general floor nurse).

Immediately present ABC information.

S-Provide a one or two liner of the current situation.

B-Present relevant background information.

A-Make an assessment about what you think is going on.

Put a name to it.

R-Make a suggestion for next steps.

Ideally, this information should take you no more than 2-3 minutes to present. In an evolving situation, this allows more time for patient evaluation and planning of next steps.
