# Supplementary material for: Simulation-Based Peer Feedback Module for Pediatric Rapid Response Team Handoffs
Source: MedEdPORTAL. 2025 Sep 5;21:11544. doi: 10.15766/mep_2374-8265.11544 (PMC12411645; doi:10.15766/mep_2374-8265.11544)
Supplement: Supplementary file 1 — RRT Facilitator Guide.docxRRT Premodule Questions.docxCase 1.docxRRT Handout.docxCase 2.docxCase 3.docxRRT Scoring Tool.docxCase 4.docxCase 5.docxRTT Postmodule Questions.docx [file mep_2374-8265.11544-s001.zip › E. Case 2.docx]

**Instructions for Use**

This simulation case is designed to be used during a 90-minute rapid response training module for pediatric and medicine/pediatric residents (PGY 1–4). Facilitators should familiarize themselves with the case details in advance and use this guide to simulate the patient scenario, provide cues at designated time points, and prompt learners as needed. This case is intended to be used in combination with the ABC-SBAR communication handout (Appendix B) and the RRT scoring tool (Appendix F). Facilitators should guide learners through assessment, intervention, and structured handoff communication using the ABC-SBAR format, followed by a debrief session.

**CASE 2:**

| SIMULATION CASE TITLE | Management of Suspected Sepsis in a Post-Traumatic Patient |
| --- | --- |
| AUTHORS | Rachael Herriman, MD, Priti Jani, MD, MPH |
| LEARNER AUDIENCE | Pediatric and medicine/pediatric residents (PGY 1–4) involved in inpatient rotation |
| PATIENT NAME | Mason |
| PATIENT AGE | 17 years |
| CHIEF COMPLAINT | Persistent fever, headache, and a new murmur |
| PHYSICAL SETTING | Pediatric inpatient unit |
| Brief Narrative Description of Case | Mason is a 17-year-old male with a history of gunshot wounds to the thoracic spine presenting with fever, headache, and a new murmur. Learners must evaluate for sepsis and endocarditis, recommend appropriate diagnostics, and communicate effectively using the ABC-SBAR framework. |
| Primary Learning Objectives | 1. Recognize clinical signs suggestive of sepsis, including fever and tachycardia, and identify a potential source of infection.  2. Recommend appropriate diagnostic studies such as CBC, CXR, and echocardiography.  3. Demonstrate a clear and prioritized handoff using the ABC-SBAR framework.  4. Collaborate with team members to develop a diagnostic and management plan, including ruling out endocarditis. |
| Critical Actions | 1. Identify fever, headache, and a new murmur as possible signs of sepsis or endocarditis.  2. Order appropriate diagnostics, including blood cultures, CBC, and echocardiogram.  3. Deliver a structured patient handoff using ABC-SBAR.  4. Monitor the patient’s response to diagnostic and therapeutic interventions. |
| Learner Preparation or Prework | 1. Review the ABC-SBAR framework.  2. Study the evaluation of febrile illnesses.  3. Understand complications in post-trauma patients |
| Section | Details |
| Initial Vital Signs | HR: 68, RR: 14, BP: 127/57, O2 sat: 94%, Temp: 38.7 |
| Overall Setting and Appearance | Patient is sitting up in bed, interactive, reporting headache and fever. Peripheral IV in place. |
| Standardized Participants | Facilitator acting as nurse, stating: 'Hi, I’m worried about Mason as he’s febrile and hypertensive. Also, I think he has a new murmur. I’m worried about sepsis.' |
| HPI | 17-year-old male with thoracic GSW to the chest and spine presenting with 7 days of headache, 3 days of fever, neck pain, and dizziness. Nursing notes a new murmur and persistent tachycardia. |
| Past Medical/Surgical History | Thoracic GSW to the chest and spine (T5-T7). |
| Medications | Vancomycin, Cefepime, Gentamycin, Morphine. |
| Allergies | Not specified. |
| Family History | Not specified. |
| Physical Exam - General | Sitting up in bed, interactive. |
| Physical Exam - Lungs | CTAB. |
| Physical Exam - Cardiovascular | 2/6 systolic murmur, capillary refill <2 sec. |
| Physical Exam - Neurological | Negative Kernig sign. |

Instructor Notes

| **Intervention / Time Point** | **Change in Case** | **Additional Information** |
| --- | --- | --- |
| 2 minutes into the case | If learners do not initiate diagnostic tests or treatment, the patient’s blood pressure begins to drop (e.g., BP decreases to 105/65). | The nurse states: “Doctor, his blood pressure is lower now. It’s 105/65. Should we be concerned?” |
| Nursing expresses concern about vital signs | The patient’s heart rate begins to increase to 85 bpm. | The nurse reports: “His heart rate is climbing. It’s now 85. He still looks uncomfortable.” |
|  | If antibiotics are not initiated… | Nurse states: “Do you think we should start antibiotics for possible sepsis? He doesn’t seem to be improving.” |
|  |  |  |

Ideal Scenario Flow

The learners enter the room to find a teenager sitting up in bed, febrile and tachycardic, with complaints of a throbbing headache and photophobia. Learners immediately connect the patient to bedside monitors and perform a thorough history and physical examination, noting a new systolic murmur. They initiate appropriate broad-spectrum antibiotics and obtain blood cultures, recognizing sepsis as a potential diagnosis. Learners order an echocardiogram to evaluate for endocarditis and complete an ABC-SBAR presentation.

Anticipated Management Mistakes

1. **Failure to connect new murmur with possible endocarditis:** Learners may miss the significance of the murmur in the context of sepsis and fever.
2. **Delay in initiating antibiotic therapy:** Some learners might not recognize the urgency of early antibiotics in sepsis management.
3. **Incomplete physical examination:** Learners may overlook the murmur or other critical findings, leading to an incomplete clinical assessment.
