# Supplementary material for: Simulation-Based Peer Feedback Module for Pediatric Rapid Response Team Handoffs
Source: MedEdPORTAL. 2025 Sep 5;21:11544. doi: 10.15766/mep_2374-8265.11544 (PMC12411645; doi:10.15766/mep_2374-8265.11544)
Supplement: Supplementary file 1 — RRT Facilitator Guide.docxRRT Premodule Questions.docxCase 1.docxRRT Handout.docxCase 2.docxCase 3.docxRRT Scoring Tool.docxCase 4.docxCase 5.docxRTT Postmodule Questions.docx [file mep_2374-8265.11544-s001.zip › F. Case 3.docx]

**Instructions for Use**

This simulation case is designed to be used during a 90-minute rapid response training module for pediatric and medicine/pediatric residents (PGY 1–4). Facilitators should familiarize themselves with the case details in advance and use this guide to simulate the patient scenario, provide cues at designated time points, and prompt learners as needed. This case is intended to be used in combination with the ABC-SBAR communication handout (Appendix B) and the RRT scoring tool (Appendix F). Facilitators should guide learners through assessment, intervention, and structured handoff communication using the ABC-SBAR format, followed by a debrief session.

**CASE 3:**

| SIMULATION CASE TITLE | Respiratory Distress in a Medically Complex Child with RSV Bronchiolitis |
| --- | --- |
| AUTHORS | Rachael Herriman, MD, Priti Jani, MD, MPH |
| LEARNER AUDIENCE | Pediatric and medicine/pediatric residents (PGY 1–4) involved in inpatient rotation |
| PATIENT NAME | Della |
| PATIENT AGE | 8 months |
| CHIEF COMPLAINT | Increased respiratory distress with RSV bronchiolitis |
| PHYSICAL SETTING | Pediatric inpatient unit transitioning to a PICU setting |
| Brief Narrative Description of Case | Della is an 8-month-old ex-preterm infant with a history of chronic lung disease, pulmonary hypertension, and RSV bronchiolitis. She presents with increased work of breathing and worsening wheezing despite current respiratory support. Learners must identify signs of clinical worsening, provide stabilization measures, communicate findings using the ABC-SBAR framework, and recommend PICU transfer. |
| Primary Learning Objectives | 1. Recognize signs of worsening respiratory distress in RSV bronchiolitis, including increased work of breathing and wheezing.  2. Synthesize clinical findings and vital signs to guide the escalation of respiratory support.  3. Demonstrate effective communication using the ABC-SBAR framework to escalate care.  4. Collaborate with team members to recommend PICU transfer and initiate further interventions as needed. |
| Critical Actions | 1. Identify increased work of breathing and wheezing as signs of respiratory distress.  2. Escalate respiratory support (e.g., increase HFNC settings).  3. Deliver a clear and structured handoff using the ABC-SBAR framework.  4. Recommend PICU transfer and ongoing monitoring. |
| Learner Preparation or Prework | 1. Review the ABC-SBAR framework.  2. Study the management of RSV bronchiolitis and indications for escalation of respiratory support.  3. Understand complications in medically complex children with chronic lung disease. |

| **Section** | **Details** |
| --- | --- |
| Initial Vital Signs | HR: 165, RR: 44, BP: 90/41, O2 sat: 100% on 6L HFNC 40% |
| Overall Setting and Appearance | Patient appears distressed, with respiratory distress and tracheal tugging. High flow nasal cannula in place. |
| Standardized Participants | Facilitator acting as nurse, stating: 'I’m worried about Della. She’s working harder to breathe, wheezing more, and showing tracheal tugging.' |
| HPI | 8-month-old ex-30-week-old infant with CLD, pulmonary hypertension, GT dependence, and septic-optic dysplasia admitted with RSV bronchiolitis. Concerns for increased work of breathing. |
| Past Medical/Surgical History | Chronic lung disease (CLD), RSV bronchiolitis, pulmonary hypertension. |
| Medications | Continuous albuterol, oxygen therapy. |
| Allergies | Not specified. |
| Family History | Not specified. |
| Physical Exam - General | Appears distressed. |
| Physical Exam - Lungs | Bilateral wheeze at bases, tracheal tugging. |

Instructor Notes

| **Intervention / Time Point** | **Change in Case** | **Additional Information** |
| --- | --- | --- |
| 2 minutes into the case | If learners do not escalate respiratory support, the patient’s oxygen saturation begins to drop to 92%. | The nurse states: “Her oxygen levels are dropping. It’s now 92%.” |
|  | If suctioning or increasing FiO2 is delayed, the patient’s respiratory rate increases to 50 breaths per minute. | The nurse adds: “Her breathing seems faster, and she’s wheezing more.” |
|  | Without intervention, the patient’s heart rate increases to 175 bpm. | The nurse urgently prompts: “Doctor, her heart rate is climbing, and she looks even more distressed. Should we call for more help?” |

Ideal Scenario Flow

The learners enter the room to find an infant in respiratory distress on high-flow nasal cannula. They increase the FiO2 to 50% and suction the airway to improve breathing. Vital signs and respiratory effort are continuously monitored. A focused history reveals chronic lung disease and RSV bronchiolitis, prompting learners to initiate continuous albuterol and steroids. Despite these measures, the patient’s respiratory status deteriorates, requiring escalation to the PICU for additional high-flow oxygen support. Resident presents ABC-SBAR during the RRT.

Anticipated Management Mistakes

1. **Failure to escalate oxygen support:** Learners may hesitate to increase FiO2 or call for additional respiratory support.
2. **Delay in administering steroids:** Some learners might not recognize the role of steroids in managing RSV bronchiolitis with chronic lung disease.
3. **Inadequate airway suctioning:** Learners may overlook this simple but crucial step in respiratory management.
