# Supplementary material for: Simulation-Based Peer Feedback Module for Pediatric Rapid Response Team Handoffs
Source: MedEdPORTAL. 2025 Sep 5;21:11544. doi: 10.15766/mep_2374-8265.11544 (PMC12411645; doi:10.15766/mep_2374-8265.11544)
Supplement: Supplementary file 1 — RRT Facilitator Guide.docxRRT Premodule Questions.docxCase 1.docxRRT Handout.docxCase 2.docxCase 3.docxRRT Scoring Tool.docxCase 4.docxCase 5.docxRTT Postmodule Questions.docx [file mep_2374-8265.11544-s001.zip › G. RRT Scoring Tool.docx]

**ABC-SBAR Pediatric Scoring Tool:**

**Instructions for Use:**
This scoring tool is intended for use during both in-person and audio-recorded simulations of pediatric Rapid Response Team (RRT) handoffs. Facilitators should complete this form while observing or listening to the resident’s simulated handoff using the ABC-SBAR framework. For audio-only assessments, omit Items 1–4. Begin timing the presentation once the resident starts the formal handoff, and record the total duration. This tool is used to provide a standardized assessment of content accuracy, organization, and brevity. It is typically used following the final simulation in the training session.

**This scoring sheet has been modified from a previous study by McCrory et al. (see below)**

**The resident will receive one point for each item 1-16. For audio scoring, please omit items 1-4 (total max score 12). Timing will begin once the resident begins a formal presentation to the team. Please be sure to include total time of presentation below.**

**Participant ID:__________________________________**

**Please mark the presentation assessment modality:**

In-person

Audio

**Handoff content:**

Item 1 (for in-person assessment only)

Item 1a: Resident accesses patient vital sign trends

Item1b: Resident evaluates labs

Item 1c: Resident evaluates, intake and output trends in the chart (for in-person assessment only)

Item 1d: Resident evaluates pediatric eCART score____

Item 2: Airway assessment (may be included in breathing assessment if air movement/breathing described as adequate) ____

Item 3: Breathing (e.g., Apnea, description of respiratory effort or air movement) ____

Item 4: Circulation ( e.g., pulses, blood pressure) ____

Item 5: Situation (reason for call) ____

Item 6: Background (e.g., reason for admission) ____

Item 7: Assessment and/or recommendation ____

**Hand off organization:**

Item 8: Airway assessment included before background, assessment or recommendation ____

Item 9: Breathing assessment included before background, assessment or recommendation ____

Item 10: Circulation assessment included before background, assessment or recommendation ____

Item 11: Situation assessment included before background, assessment or recommendation ____

Item 12: Information on airway, breathing and circulation within the first 10 seconds of the presentation ____

Item 13: Presentation completed within 30 seconds ____

**Total score in-person:**

**Total score: audio rater:______**

**Total time (in seconds) of presentation**: ______

McCrory MC, Aboumatar H, Custer JW, Yang CP, Hunt EA. “aBC-SBAR” training improves simulated critical patient hand-off by pediatric interns. *Pediatr Emerg Care*. 2012;28(6):538-543. doi:10.1097/PEC.0b013e3182587f6
