# Supplementary material for: Simulation-Based Peer Feedback Module for Pediatric Rapid Response Team Handoffs
Source: MedEdPORTAL. 2025 Sep 5;21:11544. doi: 10.15766/mep_2374-8265.11544 (PMC12411645; doi:10.15766/mep_2374-8265.11544)
Supplement: Supplementary file 1 — RRT Facilitator Guide.docxRRT Premodule Questions.docxCase 1.docxRRT Handout.docxCase 2.docxCase 3.docxRRT Scoring Tool.docxCase 4.docxCase 5.docxRTT Postmodule Questions.docx [file mep_2374-8265.11544-s001.zip › H. Case 4.docx]

**Instructions for Use**

This simulation case is designed to be used during a 90-minute rapid response training module for pediatric and medicine/pediatric residents (PGY 1–4). Facilitators should familiarize themselves with the case details in advance and use this guide to simulate the patient scenario, provide cues at designated time points, and prompt learners as needed. This case is intended to be used in combination with the ABC-SBAR communication handout (Appendix B) and the RRT scoring tool (Appendix F). Facilitators should guide learners through assessment, intervention, and structured handoff communication using the ABC-SBAR format, followed by a debrief session.

**CASE 4:**

| SIMULATION CASE TITLE | Asthma Exacerbation with Superimposed Pneumonia |
| --- | --- |
| AUTHORS | Rachael Herriman, MD, Priti Jani, MD, MPH |
| LEARNER AUDIENCE | Pediatric and medicine/pediatric residents (PGY 1–4) involved in inpatient rotation |
| PATIENT NAME | Toby |
| PATIENT AGE | 5 years |
| CHIEF COMPLAINT | Coughing, tachypnea, and oxygen desaturation with asthma exacerbation |
| PHYSICAL SETTING | Pediatric inpatient unit |
| Brief Narrative Description of Case | Toby, a 5-year-old male with a history of moderate persistent asthma, presents with worsening respiratory symptoms due to an exacerbation triggered by a viral illness and superimposed pneumonia. Learners must recognize signs of decompensation, initiate appropriate interventions, and effectively communicate findings using the ABC-SBAR framework. |
| Primary Learning Objectives | 1. Recognize clinical signs of asthma exacerbation and oxygen desaturation requiring escalation of care.  2. Implement appropriate interventions such as suctioning and adjustment of oxygen therapy. 3. Demonstrate clear and structured handoff communication using the ABC-SBAR framework. 4. Collaborate with the care team to monitor and reassess the patient’s condition after stabilization. |
| Critical Actions | 1. Identify oxygen desaturation and increased respiratory effort as signs of clinical worsening.  2. Perform suctioning and adjust FiO₂ to stabilize the patient.  3. Deliver a structured ABC-SBAR handoff to escalate care appropriately.  4. Monitor the patient’s response and provide a clear follow-up plan. |
| Learner Preparation or Prework | 1. Review the ABC-SBAR framework for structured communication.  2. Study the management of pediatric asthma exacerbations, including oxygen therapy and suctioning.  3. Understand the complications of pneumonia in patients with asthma. |

| **Section** | **Details** |
| --- | --- |
| Initial Vital Signs | HR: 90-110, RR: 30-40, BP: 111/61, O2 sat: 89-92% on 6L 40% HFNC |
| Overall Setting and Appearance | Patient is sitting in bed, playful but with respiratory symptoms and intermittent oxygen desaturations. |
| Standardized Participants | Facilitator acting as nurse, stating: 'The family called me to the room. They’re worried about his breathing.' |
| HPI | 5-year-old male with history of moderate persistent asthma and pneumonia presenting with asthma exacerbation secondary to Rhino-enterovirus. Now noted with increased coughing, tachypnea. |
| Past Medical/Surgical History | Moderate persistent asthma, superimposed pneumonia. |
| Medications | Albuterol, Ampicillin, Solumedrol. |
| Allergies | Not specified. |
| Family History | Not specified. |
| Physical Exam - General | Sitting in bed, playful. |
| Physical Exam - Lungs | Intermittent wheeze RUL, intermittent mild subcostal retractions. |
| Physical Exam - Cardiovascular | Regular rhythm. |

Instructor Notes

| **Intervention / Time Point** | **Change in Case** | **Additional Information** |
| --- | --- | --- |
| 2 minutes into the case | Oxygen saturation drops to 86% if no intervention (e.g., suctioning, FiO₂ increase) is performed. | "Doctor, his oxygen is dropping—it’s now 86%." |
| Nursing expresses concern about worsening symptoms | Tachycardia, increased work of breathing, or persistent desaturation if appropriate interventions are delayed. | *"I'm worried—his breathing still seems labored, and his retractions are worse."* |
| Participant administers bronchodilator therapy and increases FiO2 | If done promptly, patient improves with reduced wheezing and work of breathing. Delay or omission results in persistent respiratory distress. | "Should we give another breathing treatment?" |

Ideal Scenario Flow

The learners enter the room to find a young child with moderate respiratory distress and intermittent oxygen desaturations. They initiate bedside monitoring and assess the patient’s respiratory status, noting wheezing and mild subcostal retractions. Learners suction the airway, increase the FiO2 briefly to 50%, and administer albuterol. The patient’s oxygen saturation improves, and retractions resolve. A detailed history and physical examination reveal asthma exacerbation secondary to a viral URI. Learners involve respiratory therapy, and optimize asthma management with steroids and bronchodilators after delivering a successful ABC-SBAR presentation to a PICU fellow.

Anticipated Management Mistakes

1. **Failure to escalate oxygen therapy during desaturation episodes:** Learners may not recognize the need for temporary FiO2 increases.
2. **Inadequate airway suctioning:** This critical step may be overlooked, delaying symptom improvement.
3. **Overlooking the need for continuous bronchodilator therapy:** Learners might underdose or delay albuterol administration.
4. **Delayed identification of asthma exacerbation triggers:** Learners may fail to recognize the viral etiology as a contributing factor.
