# Supplementary material for: Simulation-Based Peer Feedback Module for Pediatric Rapid Response Team Handoffs
Source: MedEdPORTAL. 2025 Sep 5;21:11544. doi: 10.15766/mep_2374-8265.11544 (PMC12411645; doi:10.15766/mep_2374-8265.11544)
Supplement: Supplementary file 1 — RRT Facilitator Guide.docxRRT Premodule Questions.docxCase 1.docxRRT Handout.docxCase 2.docxCase 3.docxRRT Scoring Tool.docxCase 4.docxCase 5.docxRTT Postmodule Questions.docx [file mep_2374-8265.11544-s001.zip › I. Case 5.docx]

**Instructions for Use**

This simulation case is designed to be used during a 90-minute rapid response training module for pediatric and medicine/pediatric residents (PGY 1–4). Facilitators should familiarize themselves with the case details in advance and use this guide to simulate the patient scenario, provide cues at designated time points, and prompt learners as needed. This case is intended to be used in combination with the ABC-SBAR communication handout (Appendix B) and the RRT scoring tool (Appendix F). Facilitators should guide learners through assessment, intervention, and structured handoff communication using the ABC-SBAR format, followed by a debrief session.

**CASE 5:**

| SIMULATION CASE TITLE | Psychogenic nonepileptic seizure |
| --- | --- |
| AUTHORS | Rachael Herriman, MD, Priti Jani, MD, MPH |
| LEARNER AUDIENCE | Pediatric and medicine/pediatric residents (PGY 1–4) involved in inpatient rotation |
| PATIENT NAME | Casey |
| PATIENT AGE | 13 years |
| CHIEF COMPLAINT | Unresponsiveness and shaking movements |
| PHYSICAL SETTING | Pediatric inpatient unit |
| Brief Narrative Description of Case | Casey is a 13-year-old female with a history of recent syncope, psychiatric hospitalizations for an unspecified diagnosis, and multiple social stressors, including homelessness and concern for abuse. She was admitted for seizure-like episodes that were determined not to be seizures based on EEG and neurology evaluations. Casey underwent an extensive workup, including normal labs and a CT scan showing only an incidental sinus mucous retention cyst. The differential includes psychosocial stress-related conditions such as catatonia, functional neurologic disorder, or psychogenic nonepileptic seizures. Learners must evaluate the current presentation of unresponsiveness and shaking movements, prioritize diagnostic considerations, and communicate effectively using the ABC-SBAR framework. |
| Primary Learning Objectives | 1. Recognize clinical signs of non-epileptic events, including psychogenic or functional neurologic disorder- related symptoms.  2.Develop a differential diagnosis for unresponsiveness and shaking movements, prioritizing safety and non-invasive evaluation.  3.Demonstrate a clear and structured handoff using the ABC-SBAR framework.  4.Collaborate with team members to determine next steps for diagnostic and supportive management in a psychosocially complex patient. |
| Critical Actions | 1.Identify unresponsiveness with rhythmic shaking as possible non-epileptic events.  2.Perform a thorough physical and neurological exam, documenting findings suggestive of functional neurologic disorder or related diagnoses.  3.Communicate findings clearly during an ABC-SBAR handoff.  4.Avoid unnecessary escalation of care while ensuring the patient’s safety. |
| Learner Preparation or Prework | 1.Review the ABC-SBAR framework.  2.Study the evaluation of unresponsive pediatric patients with shaking movements.  3.Understand psychosocially driven conditions, including functional neurologic disorder and psychogenic non-epileptic seizures. |

| **Section** | **Details** |
| --- | --- |
| Initial Vital Signs | HR: 110; RR: 18; BP: 108/67; O2 sat: 98% on room air |
| Overall Setting and Appearance | The patient is lying in bed, unresponsive to commands, nail bed pressure, or sternal rub. A peripheral IV is in place. |
| Standardized Participants | The facilitator acts as a nurse, stating: “I’m worried about Casey, she’s not responsive and has shaking movements.” |
| HPI | Casey is a 13-year-old female with a significant history of recent episodes of syncope, psychiatric hospitalizations for an unknown diagnosis, and several social stressors, including a history of homelessness and concerns for abuse. She has been admitted for seizure-like episodes, which were determined not to be seizures based on EEG and neurology reports. She underwent an extensive workup, including negative labs and a CT scan showing only a sinus mucous retention cyst, likely an incidental finding. Today, Casey is unresponsive with intermittent shaking movements. |
| Past Medical/Surgical History | Recent psychiatric hospitalizations, History of syncope |
| Medications | None currently documented |
| Allergies | Not specified. |
| Family History | Not specified. |
| Physical Exam - General | Lying in bed, unresponsive to commands, nail bed pressure, or sternal rub. |
| Physical Exam - Cardiovascular | Tachycardia, well perfused, capillary refill <2 seconds |
| Physical Exam- Lungs | Clear to auscultation bilaterally |
| Physical Exam - Cardiovascular | Intermittent rhythmic jerking of the right upper extremity > left upper extremity, not suppressible.  Rigidity in bilateral upper extremities and right lower extremity with limited flexion. |

Instructor Notes

| **Intervention / Time Point** | **Change in Case** | **Additional Information** |
| --- | --- | --- |
| 2 minutes into the case | If no intervention is made, the patient remains unresponsive, and shaking movements continue. | Nurse states: “Doctor, I’m still worried. She’s not responding.” |
| Learners perform physical examination | If conducted thoroughly, findings suggestive of psychogenic non-epileptic events (wandering nystagmus, inconsistent rigidity) become more evident. | Fluctuating level of responsiveness, inconsistent movement patterns. |

Ideal Scenario Flow

Learners enter the room to find a child lying unresponsive with intermittent shaking movements. They perform a focused history and physical examination, noting wandering nystagmus and rigidity. Learners identify the unresponsiveness and shaking as non-epileptic events, likely related to psychosocial stress. They communicate findings using the ABC-SBAR framework and avoid unnecessary medical interventions. The session concludes with a discussion of psychogenic nonepileptic seizure as the leading diagnosis.

Anticipated Management Mistakes

1. **Failure to recognize non-epileptic events: Learners may escalate care unnecessarily or attempt inappropriate treatments.**
2. **Delay in recognizing psychosocial factors: Some learners might not explore the significant social history or recent psychiatric events.**
3. **Ineffective communication: Learners may struggle to organize findings and communicate them clearly using the ABC-SBAR framework.**
