# Supplementary material for: Simulation-Based Peer Feedback Module for Pediatric Rapid Response Team Handoffs
Source: MedEdPORTAL. 2025 Sep 5;21:11544. doi: 10.15766/mep_2374-8265.11544 (PMC12411645; doi:10.15766/mep_2374-8265.11544)
Supplement: Supplementary file 1 — RRT Facilitator Guide.docxRRT Premodule Questions.docxCase 1.docxRRT Handout.docxCase 2.docxCase 3.docxRRT Scoring Tool.docxCase 4.docxCase 5.docxRTT Postmodule Questions.docx [file mep_2374-8265.11544-s001.zip › J. RRT Postmodule Questions.docx]

**Rapid Response Team Post-Assessment**

Identifier (Please use the first letter of your first name, the day of birth [e.g., Feb. 7 would be 07] and the first letter of your hometown):

1. Which of the following below appropriately describes SBAR?
2. Situation, Baseline, Assessment, Referral
3. An acronym to provide structure in explaining a patient’s current status and background as well as a provider’s assessment and recommendation for what they would like to see happen for the patient
4. Sample, Background, Action, Repeat
5. An outline for a plan to transfer a patient in distress to an intensive care unit
6. Where is the appropriate location to gather (PICU and floor teams) during a RRT?
7. In the hallway outside the patient’s room
8. At a patient’s bedside
9. At the nursing station
10. In the inpatient floor team room
11. What members of the care team are required to be present at a RRT? Please select all that apply.
12. Any resident on the assigned inpatient team
13. Hospitalist attending
14. Nurse
15. PICU fellow
16. Respiratory Therapist
17. PICU Resident
18. General floor charge nurse
19. Senior resident
20. Who leads the RRT? Please choose one.
21. Hospitalist attending
22. Any resident on the assigned inpatient team
23. The nurse, if they call the RRT
24. PICU fellow
25. Senior resident
26. I have led one or more RRT(s) in the past.
27. Yes
28. No
29. I have witnessed my senior or co-resident leading a RRT.
30. Yes
31. No
32. I have used the SBAR format when leading a RRT.
33. Yes
34. No
35. N/A
36. Please rate the following on a scale from 1-5, 1= strongly disagree 2=disagree 3= unsure 4 = agree 5 = strongly agree or if not applicable, please select N/A.
37. I feel confident to lead a RRT.
38. I feel well prepared to lead a RRT.
39. A successful RRT improves patient care.
40. I have received adequate training to lead a RRT.
41. Please rate the following statements on a scale from 1-5, 1= strongly disagree, 5= strongly agree. If not applicable, please select N/A.
42. ABC-SBAR is an easy-to-use communication format for RRT events
43. ABC-SBAR is a challenging to use communication format for RRT events
44. ABC-SBAR conveys information in an easy to understand, efficient and relevant manner that will help me to improve the quality of care during a RRT event
45. ABC-SBAR conveys information in a confusing and non-efficient way and will not change the quality of care during a RRT event
46. I would recommend this format be taught to my co-residents
